# Early returning long‐distance migrant males do pay a survival cost

**DOI:** 10.1002/ece3.4569

**Published:** 2018-11-14

**Authors:** Mathilde Lerche‐Jørgensen, Fränzi Korner‐Nievergelt, Anders P. Tøttrup, Mikkel Willemoes, Kasper Thorup

**Affiliations:** ^1^ Center for Macroecology, Evolution and Climate Natural History Museum of Denmark University of Copenhagen Copenhagen Denmark; ^2^ Swiss Ornithological Institute Sempach Switzerland; ^3^ oikostat GmbH Ettiswil Switzerland; ^4^ Department of Biology Lund University Lund Sweden

**Keywords:** capture–mark–recapture, differential migration, fitness, optimal timing, protandry

## Abstract

Timing of return to the breeding area presumably optimizes breeding output in migrants. How timing affects the other components of fitness — survival, has been comparatively little studied. Returning too early in spring is expected to be associated with high mortality in insectivorous migrants when weather conditions are still unsuitable. Yet, males in particular arrive early to get access to the best territories which have been suggested to cause arrival before it is optimal for their survival. For the outward migration in autumn, timing is presumably less directly associated with reproduction and fitness and how it might affect survival is not well understood. We use data of eight songbird species ringed across Denmark to investigate how timing of return migration in spring and departure migration in autumn close to the breeding areas affects survival for short‐ and long‐distance migrants. Further, we compare survival optimum to the timing of males and females at a stopover site in Denmark in three sexually dimorphic, protandric species. We find a clear relationship between return migration and survival which differs between short‐ and long‐distance migrants: Survival decreases with date for short‐distance migrants and a bell‐shaped relationship, with low survival for earliest and latest individuals, for long‐distance migrants. In protandric species, the majority of males return before survival is optimal, whereas females on average return close to the survival optimum. The pattern of survival in relation to autumn timing is less clear, although a similar bell‐shaped relationship is suggested for long‐distance migrants. Our findings support the predicted mortality consequences of too early return to the breeding grounds and also that selection for early return in males leads to suboptimal migration timing regarding survival.

## 1 INTRODUCTION

The fitness of an individual is a result of its reproduction and survival. The importance of timing of breeding for reproduction has been documented in several studies (Daan, Dijkstra, Drent, & Meijer, 1989; Lack, 1950; Perrins & McCleery, 1989; Price, Kirkpatrick, & Arnold, 1988; Verhulst & Tinbergen, 1991) generally finding that early breeders produce most young. Migratory birds with separate breeding and non‐breeding areas have to time migration with the onset of breeding. Migrants returning early to the breeding area breed earlier (Smith & Moore, 2005; Takaki, Eguchi, & Nagata, 2001) and thereby produce more young. How adult survival, the equally important component of fitness, is affected by timing has been comparatively little studied. Presumably, the gain in reproductive success with early return is offset by a lower survival probability, but survival effects for early returning adults have only been documented in a few studies (Drent, Both, Green, Madsen, & Piersma, 2003; Newton, 2007; Takaki et al., 2001). However, especially for insectivorous species breeding at high latitudes, we expect a pronounced decrease in adult survival for very early returning individuals (Newton, 2007). In addition, we expect lower survival for late individuals, because they potentially reflect delayed individuals in poor condition (Hedlund, Jakobsson, Kullberg, & Fransson, 2014; Kokko, 1999). Furthermore, timing of migration often differs between sexes (reviewed by Morbey & Ydenberg, 2001). Within migratory songbirds, males often migrate and return before females (protandry; e.g., Rubolini, Spina, & Saino, 2004; Tøttrup & Thorup, 2008).

As migratory birds potentially compete for resources on arrival, early return to the breeding grounds to avoid competition in migratory birds might result in arrival dates that far precede the cost‐minimizing day in the absence of competition (Kokko, 1999). If only the territorial sex competes, we can expect the territorial sex, in most bird species males, to arrive earlier than the non‐territorial sex, in most bird species females. This is the “rank advantage” hypothesis (see review by Morbey & Ydenberg, 2001) which assumes indirect selection; arrival timing acts on each sex independently. Other hypotheses such as the “mate opportunity” hypothesis (Kokko, Gunnarsson, Morrell, & Gill, 2006; Morbey & Ydenberg, 2001) assume direct selection with arrival timing of one sex having fitness consequences for both sexes. Regardless of the cause of protandry, increased mortality for early arriving individuals is always assumed and actual arrival timing is a fitness trade‐off between the reproductive advantage of early arrival and the associated mortality cost.

In contrast to the situation in spring, only a few studies have investigated the effects of autumn migration timing on survival; mass mortality caused by severe weather has been documented in some cases and early cold spells in autumn prevented birds from accumulating fat reserves for migration, delaying, or even preventing departure (Newton, 2007). However, we do not know whether such rare events cause a general pattern in survival during autumn.

**Figure 1:**
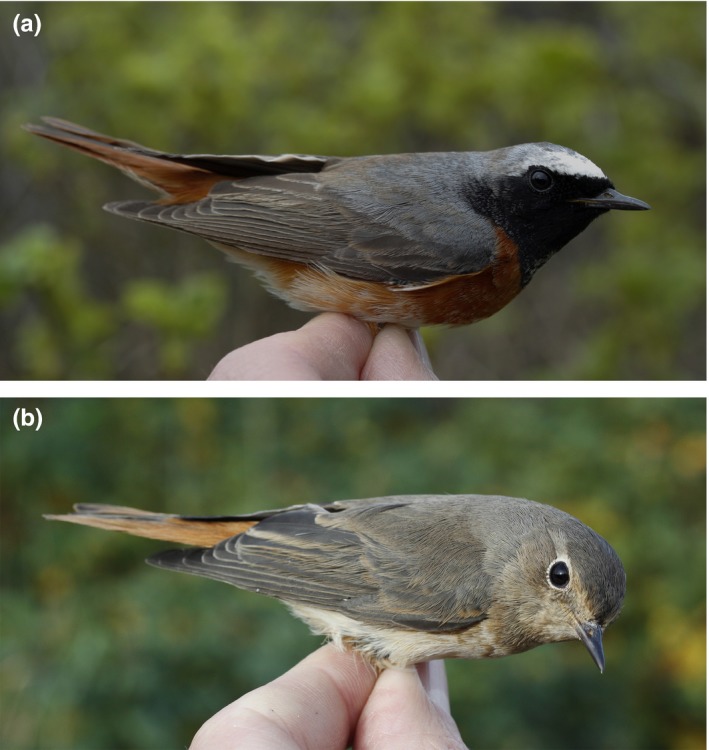
Male (a) and female (b) redstarts *Phoenicurus phoenicurus*, a sexually dimorphic, protandric (i.e., males arrive before females) species. Photographs: Henrik Knudsen

So far, the effects of migration timing on adult survival have mainly been studied theoretically, such as by game theoretic modeling. Here, we empirically investigate the effects of individual migration timing on survival for a suite of songbird species with breeding grounds primarily in North Scandinavia and Finland. Because migration timing is generally highly repeatable within an individual (Gill et al., 2014; Thorup, Vardanis, Tøttrup, Kristensen, & Alerstam, 2013), timing in one year reflects the individual timing. We use timing of migration in spring (as a proxy for breeding area arrival) and in autumn (as a proxy of breeding area departure) of individuals later recovered dead, from eight songbird species caught across Denmark during migration to estimate survival in relation to timing in spring and autumn, respectively, for long‐ and short‐distance migrants. Furthermore, we investigate whether timing at a stopover site in Denmark coincides with the highest survival and how this coincidence differs between the sexes in sexually dimorphic, protandric species (Figure 1). Given the uneven spatiotemporal sampling effort involved in collecting large‐scale ringing data, we test the robustness of our results against a range of potential confounding factors such as variation in latitudinal recovery probability as well as changes over time in recovery and survival probabilities.

## 2 MATERIALS AND METHODS

### 2.1 Modeling the effects of timing on survival

The data set consists of data from eight passerine migrant species in spring (three short‐ and five long‐distance migrants) and eight in autumn (three long‐ and five short‐distance migrants) ringed in Denmark during migration from 1950 to 2002 (Bønløkke et al., 2006; Thorup et al., 2013) and recovered dead at any time at any location (see Table 1). The birds were ringed across the country by volunteers under the national Danish ringing scheme (Copenhagen Bird Ringing Centre) and the primary breeding area is in Scandinavia and Finland (Bønløkke et al., 2006). For the recoveries included in our analyses, the date of death was recorded as accurate to the day.

**Table 1:** Species and data used in the analyses

Species	Spring											Autumn							
	Migration period	Recoveries								# migrate		Migration period	Recoveries						# migrate
		Y2	3	4	5	6	7	8	9	M	F		Y2	3	4	5	6	7	
Pied flycatcher *Ficedula hypoleuca*	April–May	14	3	3	1	1				2,625	2,538								
Common whitethroat *Sylvia communis*	April–June	7	2	2						401	204								
Lesser whitethroat *Sylvia curruca*	April–June	10	3	2	0	1													
Dunnock *Prunella modularis*	March–May	24	5	5	0	0	3	0	1	7,026		September–November	16	4	6	0	2		4,682
Willow warbler *Phylloscopus trochilus*	April–June	34	13	3	0	2				42,787		July–October	4	0	2	2			33,129
Robin *Erithacus rubecula*	April–May	42	19	8	1	1	2	1		52,375		September–November	53	17	5	0	1	1	80,914
Redstart *Phoenicurus phoenicurus*	April–June	19	8	1						3,859	4,095	July–October	4	5	2	1			1,307
Song thrush *Turdus philomelos*	April–May	41	25	7	3	2	3	1	1	8,707		August–September	84	35	16	6	3	1	21,408
Wren *Troglodytes troglodytes*												September–November	10	1					3,982
Goldcrest *Regulus regulus*												September–November	3	1	1				82,894
Reed warbler *Acrocephalus scirpaceus*												August–October	4	3	4	0	0	1	982
Total		324											303						

Note

Species included in the analyses are listed with the months of spring and autumn migration, the number of recoveries used for the survival analysis, and the total number of migrants caught in spring and autumn used in the timing analysis. Recoveries are listed as numbers of dead recoveries in a given year starting in year two (Y2). For pied flycatcher, 14 birds recovered dead in year two, three birds recovered dead in year three, three birds recovered dead in year four, and so forth.

Survival of young is often lower than adults (Grüebler & Naef‐Daenzer, 2010), but in our data, it was generally not possible to distinguish between young and adults. Instead, we excluded birds dead in the first year after ringing ensuring that only adult birds were compared. In this case, one year was subtracted from all life spans to avoid bias from the truncated distribution. We found overall same results when including birds found dead in the first year, but excluding this reduced noise in the data set (see Appendix 1 for the model results with individuals dead in the first year included).

Migration periods were defined individually for each species. Our focus was to determine overall patterns across species groups rather than species‐specific patterns. To allow for standardization of migration timing within species, we included species with at least five birds recovered after the first year. For species with a large overlap between migration and breeding (mainly hole‐nesting species such as pied flycatcher *Ficedula hypoleuca*), we chose a shorter period than the full migration period to make sure that actively breeding birds were excluded. For the species included, Denmark is near the breeding grounds (Bønløkke et al., 2006) and migration timing is highly correlated throughout northern Scandinavia (Bakken, Runde, & Tjorve, 2006). Thus, we regard capture dates as reasonable proxies for timing of return to and departure from the breeding grounds, respectively, as songbirds stopovers in Europe are generally few and relatively short, often 1–2 days (Goymann, Spina, Ferri, & Fusani, 2010; Wernham et al., 2002) but up to 15 days (Chernetsov, 2012).

We converted capture date to day of the year for all individuals (1 January = 1) and used it as a proxy for arrival (spring) or departure (autumn) day. Migration day was standardized within species subtracting the species‐specific mean and dividing by the species‐specific standard deviation. The species were divided in short‐ (European) and long‐distance (African) migrants (referred to as migration distance).

We analyze our data in a capture–mark–recapture framework using a modification of the dead recovery model for ringing data from the British Trust for Ornithology (BTO dead recovery model; Cooch & White, 2016). The BTO dead recovery model uses the time between ringing and dead recovery to estimate survival probability. The probability of finding a dead individual and reporting its ring number to a ringing scheme, hereof “recovery probability,” is thereby assumed to be independent of the age at which the individual died. The model allows estimating survival when the total number of ringed birds is unknown (Burnham, 1990) as is the case for the Danish ringing data, where only annual totals are known—not numbers ringed in specific migratory periods. Further, because survival estimates are based on the exact and known life span of the individuals, survival estimates are independent of recovery probability, that is, among‐individual variance in recovery probability does not affect survival estimates. The proportion of recoveries in the *j* ^th^ year after ringing is *S*_1_ *S*_2_ *S*_3_…*S*_*j*−1_(1 − *S*_*j*_)/(1 − *S*_1_ *S*_2_ *S*_3_…*S*_*k*_), with *k* years being the maximal duration (in years) between ringing and recovery. The number of recoveries in the *j *=* *1, …, *k* years between ringing and recovery follows a multinomial distribution with cell probabilities dependent on *j* as defined above. We used the logit link function to include a linear predictor for annual survival probability *S*_*i*_ for each individual *i*. We included spring or autumn migration date (linear and quadratic), the migration distance (short‐ vs. long‐distance migrants), and their interaction as predictors for survival probability. We further included year (linear and quadratic) in order to account for biases produced by inhomogeneous temporal distribution of our data and possible temporal changes in both survival and timing of migration. There are a number of well‐known biases in ringing and recovery data, especially given the large spatial and temporal scale of our data. However, heterogeneity of recovery probability (such as lower recovery probabilities in the northernmost latitudes compared to central Europe; Saurola, Valkama, & Velmala, 2013) is not expected to affect the robustness of our results. To confirm the lack of influence from variation in recovery probability and investigate potential effects of variation in survival, we plotted latitude against year and timing of return migration, and return migration against year (Appendix 2a–c). These plots revealed no correlations beside a weak trend toward earlier spring migration in recent years. Therefore, we included year but not latitude as a covariate in our models.

The assumption that recovery probability is independent of the age at which an individual died may be violated in our case because it is known that recovery probabilities have almost constantly declined between 1960 and 1998 (Robinson, Grantham, & Clark, 2009). Declining recovery probabilities within the life span of an individual results in an underestimation of survival. However, because we are not interested in the absolute survival estimates but in the effect of a covariate (return date) on survival, we do not expect the model assumption to bias our conclusions. As in our data set, years from ringing to recovery and year of ringing do not seem to be correlated (*r* = 0.09, *SE* = 0.04; Appendix 2e); a change in recovery probability over time is indeed unlikely to be influencing our survival estimates. Year was included as a covariate (linear and quadratic effect) in our models to account for systematic changes in survival over time, see below. Despite spring and autumn migration dates having changed over time (Tøttrup, Thorup, & Rahbek, 2006a, 2006b), such changes were only slight in our data (Appendix 2c,d). Thus, we did not include more complex interactions of survival with return date over time.

To partially pool information between the different species and to account for pseudoreplication, we included species as a random factor in the model. We modeled the intercept, and the linear and quadratic effects of day by species and assumed a normal distribution of the species‐specific deviations from the parameter means. The model was fitted to the data using Markov chain Monte Carlo simulations as implemented in JAGS that we used via the R2jags package (Su & Yajima, 2015) in R 3.2.0 (R Core Team, 2016). We used weakly informative prior distributions that should not have a recognizable effect on the results but that give a priori implausible values small probabilities as recommended by Gelman et al. (2014). We used normal distributions with means of zero and standard deviations of 5 for all the fixed effects coefficients. For the among‐species variances of the intercept, the linear and quadratic date effects, we used a folded‐t distribution with two degrees of freedom for the standard deviation (recommended by Gelman, 2006). Two Markov chains of length 10,000 were simulated with burn‐in set to 1,000 and thinning to 5. Convergence was evaluated visually and by the Brooks–Gelman–Rubin statistics (Brooks & Gelman, 1998). Spring and autumn data were analyzed separately (See JAGS code for the model in Appendix 4). A prior sensitivity analysis showed that the normal (0, 5) prior for the fixed effects had a negligible effect on the results (Appendix 5).

### 2.2 Estimating timing in relation to maximum survival

To compare the survival statistics to timing of return migration, we use percentage of ringed birds per day in the migration season on Christiansø (55.32°N, 15.2°E) from year 1976 to 1997. Christiansø is a 0.22 km^2^ rocky island located in the Baltic Sea 18 km from the nearest land source. Due to the small size of the island and limited breeding numbers, birds caught in the migration seasons are with high certainty migrants. The primary breeding grounds of these birds are North Scandinavia and Finland. We use data from Christiansø because of the long time span and a fully standardized trapping effort. The time span for the standardized trapping is shorter than our full data set and slightly biased toward late years. During the period of standardized trapping, trapping dates advanced on average by 0.26 and 0.18 days/year in spring and autumn, respectively (Tøttrup et al., 2006a, 2006b). These changes are comparatively small compared to the span of return and autumn migration timings considered here, and given that only minor changes in return time were revealed in the ringing data, we do not expect pronounced effects on our results.

The percentage is derived from the mean number of birds caught per day (calculated as birds per day summed over all years divided by total number of birds). Further, for species where data on sex are available, we derive percentages for each sex separately. To test for gender effects in our survival estimates which could confound the comparisons with timing, we ran models separately for males and females to generate gender‐specific survival curves. The gender‐specific models did not support differences among sexes in survival optimum (Appendix 3). Credible intervals (CrI) were smaller for the combined models, and these were used throughout. We compare the timing of return to the survival patterns by calculating the percentage of birds migrating before the maximum survival day for long‐distance migrants only because short‐distance migrants show no clear relationship between timing and maximum survival. We do this for each sex separately in species where males and females could be sexed.

## 3 RESULTS

### 3.1 Effects of timing on survival

**Figure 2:**
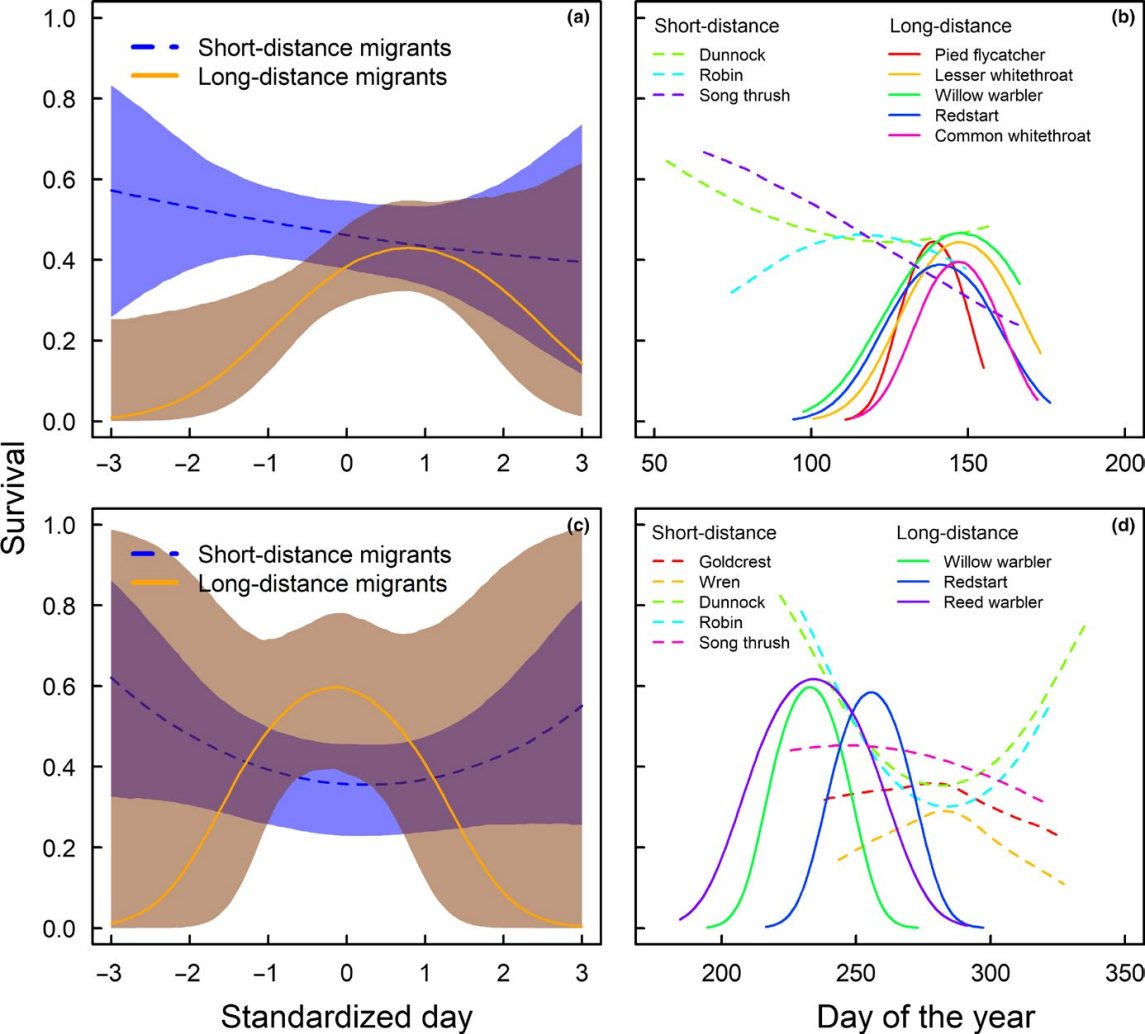
Survival in relation to return in spring (a, b) and departure in autumn (c, d). (a, c) Survival estimates for short‐ (blue dashed line is estimated with 95% credible interval shaded blue) and long‐distance migrants (orange line with 95% CrI shaded brown), respectively, as a function of standardized timing dates. (b, d) Species‐specific estimates of survival as a function of spring and autumn migration dates (day of the year) with short‐ (dashed lines) and long‐distance migrants (solid lines) indicated. In spring (a, b), we find a strong relationship between survival and timing of return; long‐distance migrants have a survival optimum on an intermediate return date and short‐distance migrants show decreasing survival from early to late return dates. In autumn (c, d), the pattern is less clear, but there are indications of a survival optimum for long‐distance migrants

We find that short‐distance migrants had the highest survival if returning early and their survival decreases with migration day in spring. Long‐distance migrants returning early have low survival, and individuals migrating slightly later than the species‐specific average return time have highest survival. For late returning long‐distance migrants, the mean survival seems to decrease again. However, the uncertainty of these survival estimates is large so that we do not know whether the survival patterns for late returning individuals differ between long‐ and short‐distance migrants (Figure 2a).

For the North European migrants, we find that maximum survival is associated with spring migration dates around 19 May for pied flycatcher, 21 May for redstarts *Phoenicurus phoenicurus*, 26 May for common whitethroat *Sylvia communis,* and 20 May for lesser whitethroat *Sylvia curruca* which is before the maximum for willow warbler *Phylloscopus trochilus* on 28 May (Figure 2b). There are also indications of a survival optimum for robins *Erithacus rubecula* around 1 May, whereas survival in song thrush *Turdus philomelus* and dunnock *Prunella modularis* was the highest early in the season.

In autumn, short‐distance migrants migrating early and late have the highest survival while long‐distance migrants show the opposite pattern with a survival optimum on intermediate dates. However, 95% CrIs for long‐distance migrants are large. Hence, it is difficult to determine the survival at the beginning and end of the migration periods (Figure 2c). Short‐distance migrant species show varying patterns with robin and dunnock driving the pattern of high survival for early and late autumn migration days found in the initial model (Figure 2d).

### 3.2 Timing in relation to maximum survival

**Figure 3:**
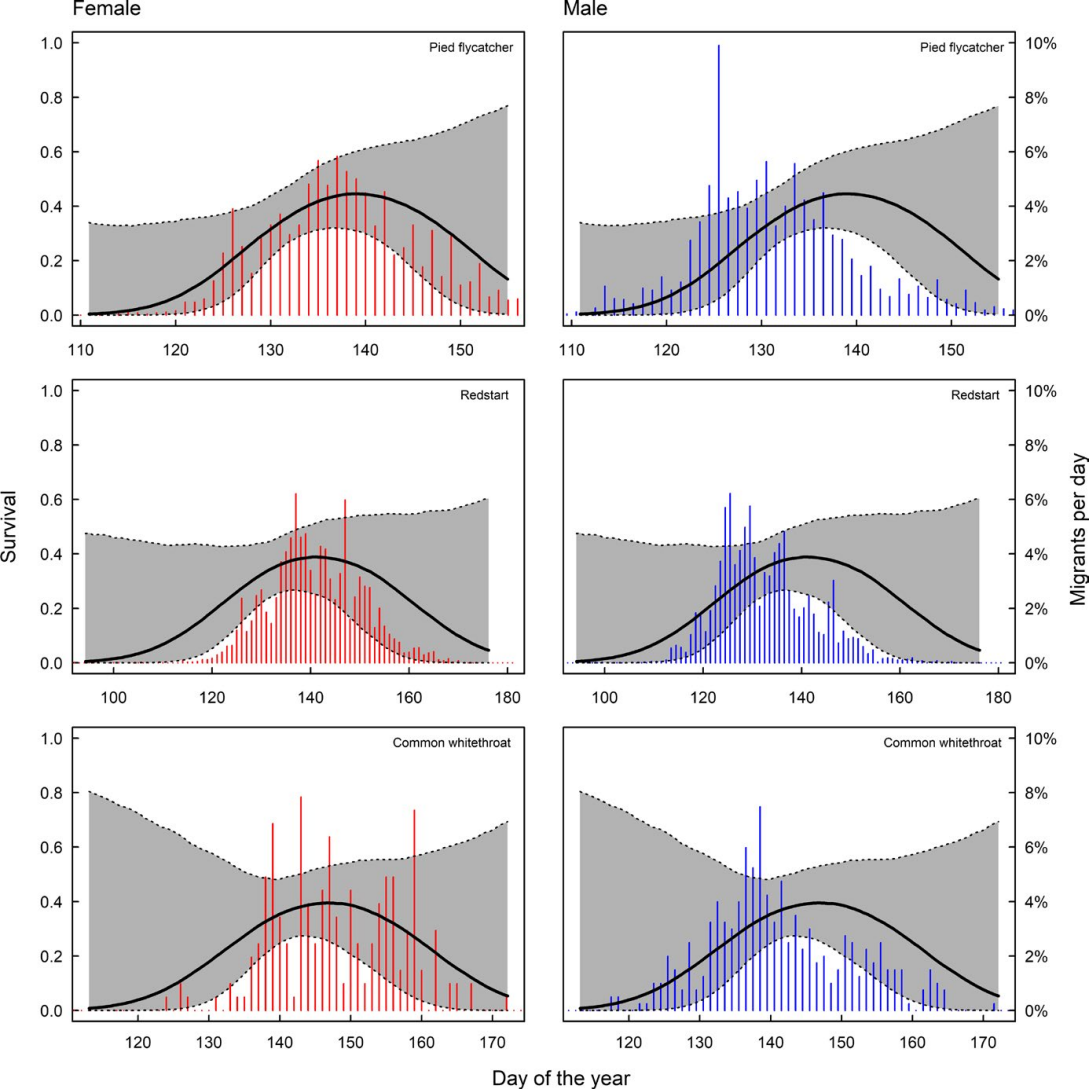
Return of males and females, respectively, in relation to survival. The bars are the proportion of males (blue) and females (red) migrating per day in spring. For pied flycatchers, 81% of the males and 56% of the females migrated before the survival optimum, for redstarts, 79% of males and 51% of the females, and for the common whitethroat, 72% of the males and 46% of females migrated before the survival optimum

**Figure 4:**
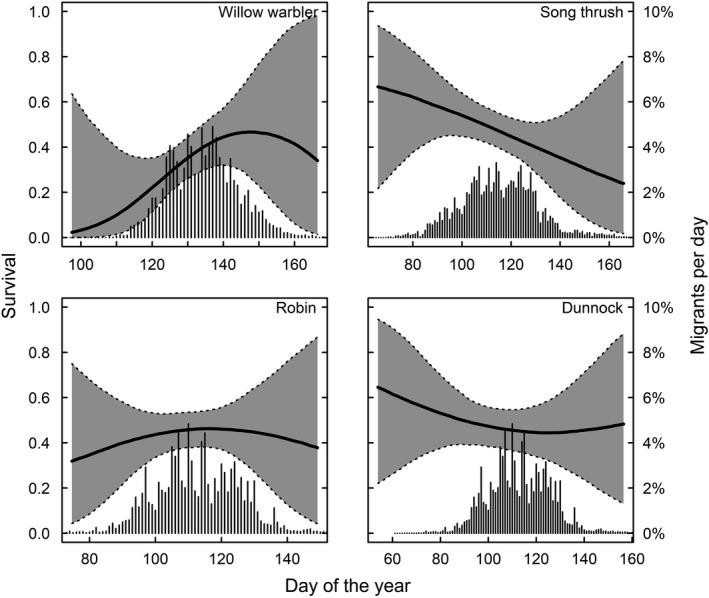
Survival in relation to return timing (spring) for one long‐distance (willow warbler) and three short‐distance migrants that could not be sexed. The bars show the proportion of birds migrating per day in spring. For willow warblers, 92% of the birds arrived before the survival optimum

**Figure 5:**
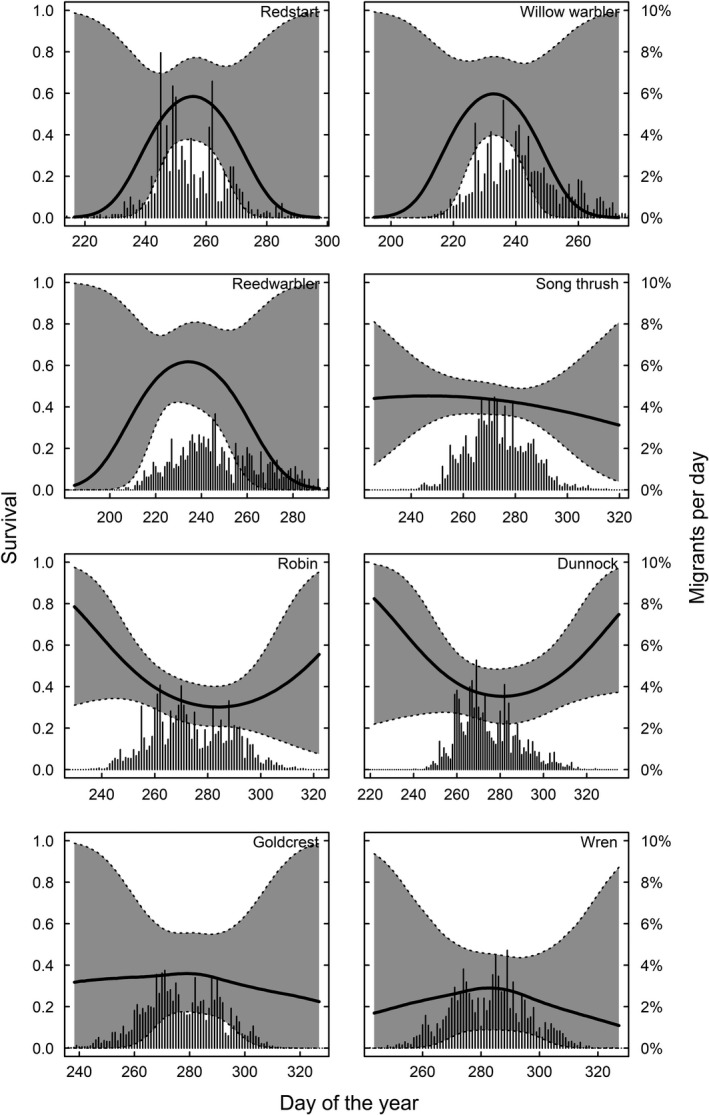
Survival in relation to departure timing (autumn). In redstarts, 50% of the birds depart before maximum survival day (8 September); reed warblers 19% depart (17 August); and willow warblers 23% (19 August)

In long‐distance migrants, peak spring migration occurs before the date of maximum survival. In species where males and females can be separated, only males arrive before the optimum and females come close to coincide with maximum survival (Figure 3). We find that 81% of the males and 56% of the females pass before maximum day of survival in pied flycatchers, for redstarts 79% of males and 51% of females pass before maximum survival and for common whitethroat 72% of males and 46% of females pass before the survival maximum (Figure 3). Timing in relation to survival in spring for species that could not be sexed is shown in Figure 4, and timing in relation to survival in autumn is shown in Figure 5.

## 4 DISCUSSION

We find a strong relationship between timing of return migration in spring and annual adult survival. The pattern differs between short‐ and long‐distance migrants; long‐distance migrants have a survival optimum on an intermediate migration date, and short‐distance migrants show decreasing survival from early to late dates. In long‐distance migrants, the majority of males return before the survival optimum while females return on average at dates associated with the highest survival.

It is a common assumption that birds need to time their migration to return early to maximize their chance of reproducing successfully (Both, Bowhuis, Lessels, & Visser, 2006; Smith & Moore, 2005; Takaki et al., 2001) yet not too early because they risk arriving on the breeding grounds when environmental conditions are still too harsh (Drent et al., 2003; Newton, 2007). The latter is clearly reflected in the survival pattern for long‐distance migrants in this study, but not for short‐distance migrants.

The low survival we find for early returning long‐distance migrants could be due to cold spells after arrival, causing starvation when food becomes unavailable either because of snow cover or because insects become dormant at low temperatures (Newton, 2007). Long‐distance migrants migrate within a short time frame in spring with little year‐to‐year variation in arrival dates (Hagan, Lloyd‐Evans, & Atwood, 1991; Mason, 1995) indicating a high level of endogenous control of timing of the return to breeding grounds. It is possible that the long‐distance migrants do not fully adjust migration timing to occurring weather conditions perhaps due to a strong endogenous clock (Bussiere, Underhill, & Altwegg, 2015). In contrast, we find that short‐distance migrants returning early have the highest survival which might indicate that short‐distance migrants are less strongly affected by harsh weather conditions and that fit individuals gain more and risk less by arriving early. This hypothesis is supported by the fact that some short‐distance migrants stay at the breeding ground during winter (Lack, 1944; Partecke & Gwinner, 2007). But it is also likely that they are better at adjusting timing of arrival to occurring weather condition because they winter closer to the breeding area as indicated in other studies where they found more flexible arrival dates and arrival over a larger time span in short‐distance than long‐distance migrants (Hagan et al., 1991; Mason, 1995). The indication of low survival on late dates that we find for both long‐ and short‐distance migrants probably reflects individuals in poor body condition arriving late in the season (Hedlund et al., 2014; Kokko, 1999).

Early assumptions of order of returning to the breeding grounds were that it was a direct reflection of fitness where fittest individuals arrived first because they were able to outweigh the costs of arriving early (Kokko, 1999). If we only consider the fitness parameter survival, this seems to be the case for short‐distance migrants where we find decreasing survival with return date which represents an “honest arriving order.” However, for long‐distance migrants where the first returning individuals suffer from lower survival, the reproductive benefits would need to exceed the disadvantages of low survival to hold true (Alerstam, 2006). It has been suggested that timing and order are not a direct reflection of fitness. Instead, individuals consider the availability of resources (e.g., territories or mates) and timing of conspecifics in arrival timing decisions (Kokko, 1999; Sirot & Touzalin, 2014). We find that males return before the survival optimum while female return date seems to coincide with maximum survival.

The separate survival curves we derive for each sex, which shows that optimal survival date corresponds between the two sexes, are based on a small sample size, and hence, we cannot be completely certain that the survival optimum for males and females is the same. Yet, if the optimum for males in our analysis is earlier, the female optimum would be later and females would return before their survival optimum. We consider this less likely as the low survival for early arriving individuals is most likely due to lack of food or cold spells (Newton, 2007), and this would probably affect both sexes and be less likely to occur much later in the season for one sex than the other. This is supported by a study by Saino et al. (2010) where they found no evidence that the larger body size in males is an adaptation to resist harsh conditions early in spring.

In autumn, we in general find the survival pattern to be less clear; long‐distance migrant shows higher survival at intermediate dates which is not unlikely. Migrating late in the season can clearly be disadvantageous due to the risk of encountering harsh weather which can unable the birds to fuel for migration or simply cause mortality (Newton, 2007) and early individuals can reflect unpaired males or failed breeders (Kjellén, Hake, & Alerstam, 2001; J. Ouwehand, unpublished) which have been shown to be in poorer condition than breeding individuals in some species (Chastel, Weimerskirch, & Jouventin, 1995). For short‐distance migrants, we find survival to be the highest for early and late timing, but this does not seem to be a general pattern as it is only present in robin and dunnock. The varying survival patterns for short‐distance migrant species indicate a less strong selection pressure on timing in autumn.

Returning early has a clear impact on the chance of surviving in long‐distance migrants yet in particular males compete to arrive at the breeding ground first. Timing of spring arrival has advanced during the last decades (Gill et al., 2014; Hedlund et al., 2014; Jonzén et al., 2006; Mason, 1995; Tøttrup et al., 2006a) maybe as a consequence of this mechanism. However, advancement of one event in the year cycle can cause mismatch between year events and environment as is seen with breeding and peaks of food availability (Both et al., 2006). This is a major concern especially for migratory birds, because changes in climate are happening at different pace around the globe (Both & Visser, 2001) causing great uncertainties about future climate scenarios. Besides shedding light on the effect of migration timing on adult survival, this study adds survival to the equation of how climate changes are affecting fitness in migratory birds.

## CONFLICT OF INTERESTS

The authors have no conflict of interests to declare.

## DATA ACCESSIBILITY STATEMENT

The ringing data are archived by Copenhagen Bird Ringing Center.

## AUTHOR CONTRIBUTIONS

KT conceived the study. KT, MLJ, FKN, APT, and MW designed the study. MLJ and FKN analyzed the data. All authors interpreted the results. KT, MLJ, and FKN wrote the manuscript; other authors provided editorial advice.

## APPENDIX 1

### APPENDIX 1

Survival in relation to return and departure when birds dead within the first year are included. Survival in relation to arrival in spring (a, b) and departure in autumn (c, d). (a, c) Survival estimates for short‐ (blue dashed line, 95 % credible interval shaded blue) and long‐distance migrants (orange, 95 % CrI shaded brown), respectively, as a function of standardized timing dates. (b, d) Species‐specific estimates of survival as a function of arrival and departure dates (day of the year) with short‐ (dashed lines) and long‐distance migrants (solid lines) indicated. In spring (a, b), we find a strong relationship between survival and timing of arrival; long‐distance migrants have a survival optimum on an intermediate arrival date, and short‐distance migrants show decreasing survival from early to late arrival dates. In autumn (c, d), the pattern is less clear, but there are indications of a survival optimum for long‐distance migrants

### APPENDIX 2

Robustness of results. Evaluation of potential confounding factors such as variation in latitudinal recovery probability and changes over time in recovery and survival probabilities. (a) Latitude of recovery against recovery for birds ringed in spring. (b) Recovery latitude against day of ringing for birds ringed in spring. (c) Year of recovery against day of ringing for birds ringed in spring. (d) Year of recovery against day of ringing for birds ringed in autumn. (e) Changes in recovery probability or survival over time for birds ringed in spring indicating no correlation between years from ringing to recovery and year of ringing (black squares are yearly averages). Each point represents a recovery in all panels

### APPENDIX 3

Sex‐specific survival curves. Survival as function of return date for females (red) and males (blue). In pied flycatchers (top), there was a small difference between males and females in optimal survival date. Yet, males arrive before their optimal survival date. For redstarts (middle) and whitethroats (bottom), optimal survival dates for males and females were similar

### APPENDIX 4

JAGS code

#### APPENDIX 5

Sensitivity of model results to the prior distributions of the fixed effects

To assess how strongly the prior is affecting the results, we re‐fitted the model using two different sets of prior distributions. We first increased the precision of the (normal) prior by using a standard deviation of 0.8 instead of 5 and then decreased the precision by using a standard deviation of 10 instead of 5.

The increase in the precision (i.e., a decrease in the standard deviation from 5 to 0.8) did only very slightly affect the parameter estimates, in that the fixed effects were slightly shrunk toward zero and standard errors became smaller. However, the shrinkage toward zero was compensated by a slight increase in the species‐specific random effects so that species‐specific fitted values were not noticeably affected.

The decrease in the precision (i.e., an increase in the standard deviation from 5 to 10) did not noticeably affect the results.
